# Distinct functional connectivity patterns in myalgic encephalomyelitis and long COVID patients during cognitive fatigue: a 7 Tesla task-fMRI study

**DOI:** 10.1186/s12967-026-07708-y

**Published:** 2026-01-20

**Authors:** Maira Inderyas, Kiran Thapaliya, Sonya Marshall-Gradisnik, Leighton Barnden

**Affiliations:** National Centre for Neuroimmunology and Emerging Diseases, Griffith University, Southport, QLD Australia

**Keywords:** Long COVID, ME/CFS, Functional connectivity, 7 Tesla, Task functional MRI

## Abstract

**Background:**

Myalgic Encephalomyelitis/Chronic Fatigue Syndrome (ME/CFS) and long COVID are chronic debilitating illnesses featuring fatigue, post-exertional malaise (PEM) and neurocognitive deficits. Temporal correlation of neural activity between distinct brain regions, also referred to as functional connectivity (FC), can provide insights into how brain networks coordinate, at rest or during task. Therefore, we explored intrinsic FC correlates of cognitive fatigue in ME/CFS and long COVID patients during two Stroop-colour-word paradigms on 7 Tesla fMRI.

**Methods:**

450 sagittal volumes were acquired from seventy-eight participants: 32 patients with MECFS (pwME/CFS); 19 long COVID (pwLC) and 27 healthy controls (HC) during performance of baseline or *Pre* (before/during fatigue build-up) and repeat *Post* (fatigue set-in) Stroop tasks. Structural and functional data were analysed using the CONN toolbox.

**Results:**

Regions of interest (ROI-to-ROI) analysis revealed significantly increased FC in subcortical regions in HC for *Pre* vs *Post*. Relative to HC, pwLC showed significantly reduced FC between nucleus accumbens and vermis 3 *(p = 0.02)* in *Pre* and increased FC in the prefrontal cortex and hippocampus *(p = 0.02)* in *Post.* pwME/CFS showed a significantly increased FC between the left cuneiform nucleus and right medulla *(p = 0.03)*. Compared to HC, reduced FC was significant in pwLC during *Pre,* and between medulla and hippocampus *(p = 0.04)* and between nucleus accumbens and vermis (*p = 0.001)* during *Post.* Aberrant FC was significant for pwME/CFS in core networks during *Pre.* Core network FC to the cerebellum, amygdala, caudate and red nucleus correlated with symptom scores for cognition in both pwME/CFS and pwLC. Hippocampus and cerebellar FC correlated with duration of illness in pwME/CFS.

**Conclusions:**

Our findings of reduced dopaminergic hippocampal-nucleus-accumbens connectivity imply blunted motivation and cognition. Extensive FC differences in subcortical and core networks in patient cohorts were detected relative to an increased FC in HC. High regional communication indicative of greater task engagement by HC was distinctive while FC differences in ME/CFS and long COVID patients indicated reduced and dysregulated regional coordination that may serve as candidate biomarkers of symptomatology in long COVID and ME/CFS.

**Supplementary Information:**

The online version contains supplementary material available at 10.1186/s12967-026-07708-y.

## Introduction

Myalgic Encephalomyelitis/Chronic Fatigue Syndrome (ME/CFS) is a chronic, debilitating condition characterised by unexplained fatigue that persists for at least 4 to 6 consecutive months and is not ameliorated after rest [1–3]. Recently, post-COVID condition, or long COVID has been reported as an ME/CFS-like condition that also presents with persistent fatigue and cognitive complaints [4] sharing significant overlap with the clinical presentation of ME/CFS [5]. Evidence suggests that both illnesses evolve into chronic multisystemic dysfunction following an initial infection [6, 7] and share hallmark symptoms of persistent physical and mental fatigue, neurocognitive deficits, brain fog, autonomic and sensory dysfunctions [8, 9]. Post-exertional malaise (PEM) is key feature in both [9, 10]. Diagnostic challenges are compounded since the two conditions are regarded as “diagnoses of exclusion”, where diagnosis is made by ruling out other conditions with similar symptoms while meeting diagnostic criteria for ME/CFS [1–3, 11] and long COVID [12, 13]. The aetiology underlying these conditions remains unresolved with an ambiguous prognosis [14, 15].

Fatigue and post-exertional malaise (PEM) are the most common symptoms in patients with ME/CFS (pwME/CFS) and long COVID (pwLC), resulting in compromised daily functioning [16]. Neurologically, ME/CFS and long COVID individuals present abnormalities indicating the key role of the central nervous system (CNS) and the autonomic nervous system (ANS) [17–19]. These may stem from reduction in cerebral blood flow (CBF) [20, 21], alterations in limbic axes [22–25], and/or structural brain abnormalities [26–34]. Such alterations lead to a constellation of neurological symptoms that converge to cognitive dysfunction, neural impairment and exhaustion in both conditions.

Blood oxygen-level-dependent (BOLD) functional magnetic resonance imaging (fMRI) is widely used to study the neural correlates of cognitive processes by tracing activation, deactivation and connectivity within brain regions [35, 36]. The correlation between BOLD time-series across two brain regions measures functional connectivity (FC) between them [37]. Such connectivity between regions can then be used to test the neural consequences during acute neurocognitive challenges to infer the underlying neural mechanisms. The classic Stroop colour-word naming task is one such cognitive test that is extensively used to assess the ability to inhibit cognitive ‘interference’, a phenomenon that arises when processing information related to a feature of a particular stimulus disrupts the simultaneous processing of another feature of the stimulus [38]. Neural exertion response may be expressed as an aversion towards the task and can indicate neurocognitive fatigue resulting in problem-solving and task execution difficulties [39–41], changes in task-region recruitment [42], region disengagement [43] and reorganisation of regional connectivity [34, 44–46].

Recently, we reported that pwME/CFS and pwLC had significantly delayed response times to the Stroop stimuli when compared with HC [10]. This delay in response times actively distinguished information conflict and how participants adjusted information processing dynamically to optimise performance when presented with conflicting information. In ME/CFS, slowed information processing may most likely be a consequence of the structural and functional abnormalities in the cingulate cortex, cerebellum, cerebellar vermis and deep grey matter regions [29, 42, 47–50]. The brainstem and the nuclei of the basal ganglia: the thalamus, pallidum, putamen, hippocampus [28, 51–61] have all been implicated in ME/CFS and found to be correlated with cognitive impairment [56]. Likewise, for both ME/CFS and long COVID, altered FC has been reported within the three “core intrinsic networks” of the brain: the salience (SN), the default mode network (DMN) and the central executive network (CEN) [35, 50, 59, 62–64] with persistent FC deficits following recovery from COVID-19 infection [65–68].

Since mental fatigue and exhaustion can be driven by dysfunctional brain regions, understanding their neurobiological bases is critical. Together with the limited information available on functional connectivity in ME/CFS and long COVID, we aim to understand the FC patterns in both illnesses and how they differ from FC in healthy individuals during mentally demanding tasks. To the best of our knowledge, this is the first study comparing FC between pwME/CFS and pwLC using 7Tesla (7T) fMRI and their differences from a healthy population. Our targeted regions of interest (ROIs) included the triple network regions of the DMN, SN and CEN, respectively, involved during resting state, salient internal and external stimuli and task engagement [69]. These intrinsic networks operate in conjunction with subcortical regions to form a cortical-subcortical organisation of functional networks [70–73]. Structures of the frontal, limbic, striatum, basal ganglia and parietal areas may cluster to constitute fatigue networks in the brain. This underpins key functions of cognitive and motivation systems of the brain [74] that are likely to be implicated in cognitive, somatosensory and emotional symptoms responsible for mental fatigue [75]. Since ME/CFS and long COVID present characteristic features of brain fog, cognitive fatigue and neurocognitive deficits, we reasoned that these regions would be implicated during the fatiguing Stroop task.

Therefore, the present study aimed to investigate the effects of cognitive exertion-induced by the Stroop-colour-word task on differential FC patterns in pwLC, pwME/CFS and healthy controls (HC). Using 7T fMRI, we intend to capture subtle signals from deeper cortical and subcortical layers. Our goals were threefold; (1) to identify within-group FC pattern differences in cortical and subcortical ROIs during two task fMRI sessions, i.e., the initiation or early stages of interference-induced cognitive fatigue (*Pre*) and when interference-induced cognitive fatigue sets in (*Post*); (2) to perform between-group comparisons to examine the FC differences between pwME/CFS and pwLC with HC; and (3) to explore FC associations with the disease course via cognitive symptom severity measures in both patient groups.

## Methods

### Participant recruitment

This study was approved by the Griffith University Human Research Ethics Committee (2022/666). This cross-sectional study was conducted at the National Centre for Neuroimmunology and Emerging Diseases (NCNED) on the Gold Coast, Queensland, Australia, with written informed consents obtained from all participants. Nineteen pwLC fulfilling the WHO criteria for post-COVID-19 according to the Delphi consensus [12], thirty-two pwME/CFS fulfilling the Canadian Consensus Criteria (CCC) and/or International Consensus Criteria (ICC) [2, 3] and twenty-seven HC without any chronic health conditions or evidence of underlying illness were recruited for the study. The inclusion criteria for participants were the same as that in our previous publication [61] where all participants were aged between 18 and 65 years. Individuals with any chronic malignancies, autoimmune, neurological or psychological, or cardiovascular diseases, pregnancy and/or breastfeeding were excluded from the study. Participants were requested to complete the online research registry questionnaires which incorporated the CDC chronic fatigue syndrome (CFS) inventory and a series of validated quality of life (QoL) measures that could be accessed via Research Electronic Data Capture (REDCap). In particular, we sought information on each participant’s history of COVID-19 infection, or multiple infections, approximate dates of infection(s) and whether the diagnoses were made through COVID-19 polymerase chain reaction (PCR) test or a Rapid Antigen Test. We also acquired the duration of illness and cognitive symptom scores from pwME/CFS and pwLC (see Table 1 for demographic information).

**Table 1:** Demographic information for pwLC, pwME/CFS, and healthy controls (HC), number of females and males in each group, number of females/males (*f/m*), their mean and standard deviation (SD) for age, and clinical measures is provided. (see Supplementary Table 1 for age, sex and symptom scores for each participant included in the final analysis)

Cohorts	f/m	Age	Symptom Severity Scores		Two tailed unpaired T-test p value		
			Duration of Illness	WHODAS Cognitive	Age	Duration of Illness (years)	WHODAS Cognitive
pwLC (*n* = 17)	12/5	46.20 ± 12.62	0.8± 0.6	34.57 ± 17.1	*0.4*^*a*^	*p = <0.001*^*a*^	*p = 0.002*^*a*^
pwME/CFS (*n* = 27)	19/8	42.9 ±10.5	12.7 ±11.0	53.4±15.1	*0.003*^*b*^		*p = <0.001*^*b*^
HC (*n* = 24)	15/9	34.75 ± 8.39	*N/A*	4.6±7.1	*0.003*^*c*^		*p = <0.001*^*c*^

*a = pwLC vs pwME/CFS, b = pwME/CFS vs HC, c = pwLC vs HC. N/A denotes not available*

### Clinical scores

Duration of illness for pwLC and pwME/CFS post onset of symptoms was obtained from self-reported data and formal diagnoses by the medical doctors. The WHO Disability Assessment Schedule (WHODAS 2.0), which uses seven subscales to assess quality of life measures, where each subscale has a minimum possible score of 0 and a maximum of 100, was used to assess cognition measures in ME/CFS and long COVID. As reported in Table 1 and Supplementary Table 1, these scores are directly proportional to disability and/or dysfunction [76].

### MRI acquisition

We acquired functional and structural MRI data using the same protocol as per our previous study [61]. All 78 participants underwent scanning on a 7T whole-body MRI scanner (Siemens Healthcare, Erlangen, Germany) using a 32-channel head coil (Nova MRI Wilmington NC, USA). Anatomical sagittal T1-weighted images were acquired using a Magnetization Prepared 2 Rapid Acquisition Gradient Echo (MP2RAGE) sequence with repetition time (TR) = 4,300 ms, echo time (TE) = 2.45 ms, first inversion time (TI1) = 840 ms, second inversion time (TI2) = 2,370 ms, first flip angle (FA1) = 5°, second flip angle (FA2) = 6°, and resolution = 0.75 mm^3^ with matrix size = 256 × 300 ×320. Sagittal functional volumes for two fMRI sessions (450 in total) were acquired using a multiband echo-planar imaging (EPI) pulse sequence developed at the University of Minnesota (Auerbach et al., 2013) during which the participants responded to the Stroop colour-word task stimuli. The fMRI acquisition parameters were: TR = 2000 ms, TE = 22.4 ms, flip angle = 70°, multi-slice mode = Interleaved, acquisition matrix 192 × 192 and voxel size = 1.25 mm^3^.

### The Stroop colour-word paradigm

**Fig. 1:**
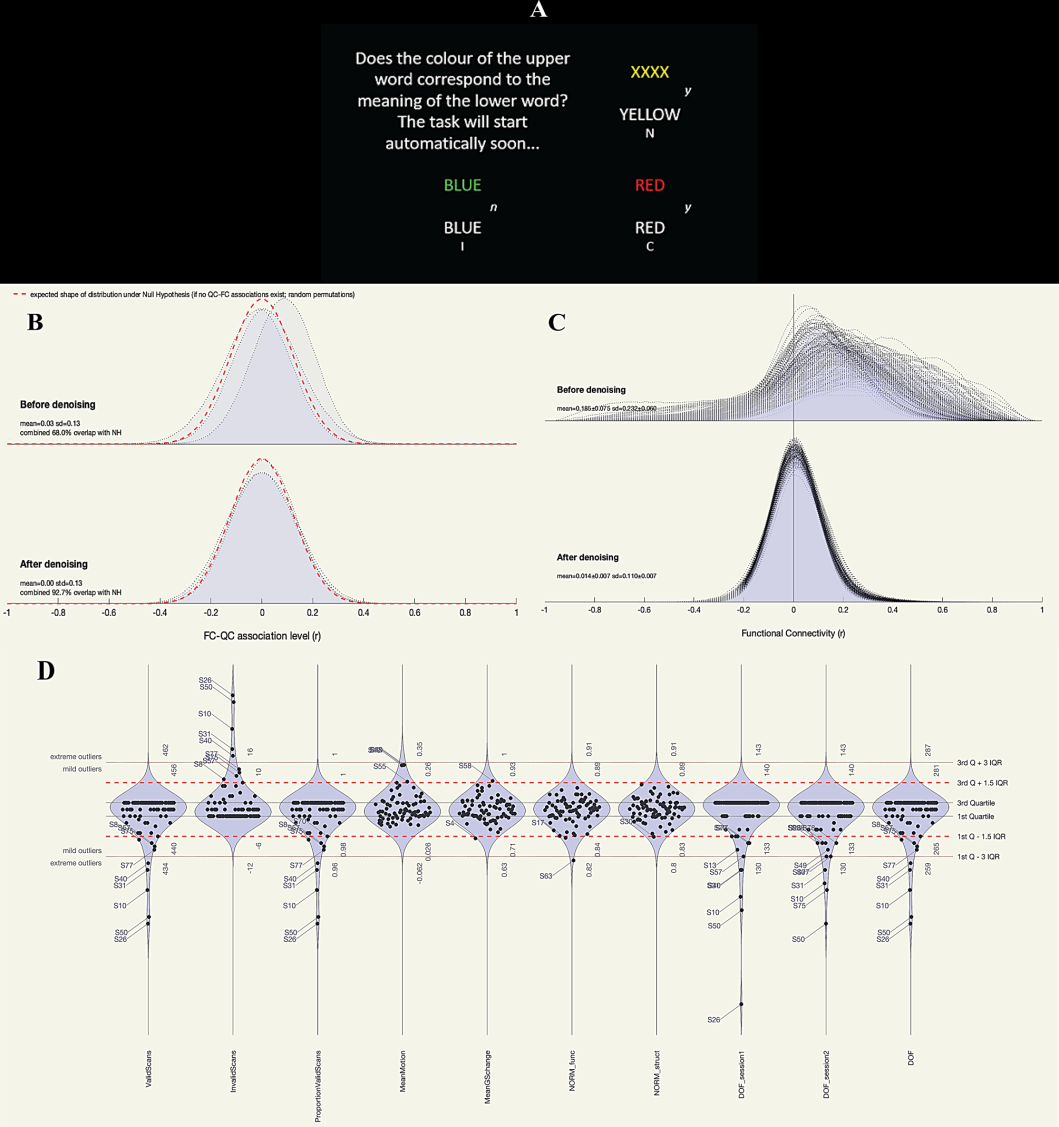
(**A**) Illustration of the Stroop colour-word task with four examples displayed on the screen to the participants. Top left: instructions, top right: neutral (N); lower left: incongruent (I); lower right: congruent (C); on the right *y* indicates answer *yes*, *n* indicates answer *no* to instructions. (**B**) Quality control (QC) measures across all participants before denoising (*n* = 78) and after denoising excluding outliers (*n* = 68); QA denoising: distribution of QC-FC associations; (**C**) distribution of functional connectivity values; and (**D**) QA variables: distribution of subject-level QC measures (*BOLDstd, standard deviation of the BOLD signal; DOF, degrees of freedom; GCOR, global correlation; IQR, interquartile range; NORM*_*func*_, *MNI-space template to functional overlap; NORM*_*anat*_, *MNI-space template to anatomical overlap; PVS, proportion of valid scans)*

Two sessions of task fMRI were performed during a cognitively challenging Stroop paradigm. Session 1 of the fMRI task targeted at the initiation or buildup of cognitive fatigue and is referred to as the pre-interference-induced cognitive fatigue condition or *Pre* throughout the manuscript. *Pre* consisted of 60 Stroop stimuli, session 2 commenced 90 seconds after the end of session 1 where cognitive fatigue sets-in following *Pre* and is referred to as post-interference induced cognitive fatigue condition (*Post*). As in our previous publication [61], participants were presented with two coloured words on the screen where upper word was read as either ‘Blue’, ‘Red’, ‘Yellow’, ‘Green’ or ‘XXXX’ and coloured in red, green, blue or yellow ink against a black background. Lower words read as ‘Blue’, ‘Red’, ‘Yellow’ or ‘Green’ and were inked in white against a black background, (see Fig. 1–A). Participants were instructed to decide whether the colour of the upper word agreed with the meaning of the lower word and respond with a ‘*yes*’ or ‘*no*’ by pressing one of the two buttons on the handpiece provided inside the scanner. By naming the colour of the upper word, a voluntary effort was required to choose the colour, while ignoring the word itself which is contrary to the automatic association between the idea and the name (saying ‘Green’ to the word ‘Green’), known as conflict or interference. The paradigm was divided into four conditions: 1) neutral (upper word ‘XXXX’), 2) incongruent (upper word inked in a different colour from its meaning), 3) congruent (upper word inked in the colour corresponding to its meaning), and 4) an idle period or the “rest” between a trial response and the next trial onset during which a stationary cross appeared on the screen for a randomised period between 3 and 12 seconds. Forty percent of trials were incongruent, 30% were congruent and 30% were neutral and the average inter-stimulus time was 10.5 seconds. Both runs consisted of 120 stimuli in total (60 per session). Two fMRI tasks lasted for 7.5 minutes each, separated by 90 seconds.

### Regions of Interest (ROI)

The default atlas from the functional connectivity CONN toolbox was used during the analysis for regions from the default mode and the salience networks, cerebellum and deep grey matter. CONN uses by default a combination of the Harvard-Oxford atlas and the AAL atlas. Brainstem regions that we used in the analyses were extracted using Statistical Parametric Mapping (SPM-12). List of 67 ROIs used in the analysis with their lateralities have been included (Supp. Table 2 and Supp. Fig. 1).

### Preprocessing

Results included in this manuscript were acquired from fMRI data analyses performed using CONN [77] (RRID:SCR_009550) release 22.v2407 [78] and SPM [79] *(RRID:SCR_007037) release 12.7771* running in MATLAB 2019b (The MathWorks, Inc.). MRI data processing was performed as in our previous publication [61]. Functional and anatomical data were pre-processed using a modular preprocessing pipeline [80] including realignment with correction of susceptibility distortion interactions, slice timing correction, outlier detection, direct segmentation and MNI-space normalization, smoothing, and removal of initial scans. The first 5 scans in each functional run were removed to ensure that the data reached a steady state and to eliminate artifacts. Functional images were realigned using SPM realign & unwarp procedure [81], where all scans were registered to a reference image (first scan of the first session) using a least squares approach and a 6-parameter (rigid body) transformation [82], and resampled via B-spline interpolation to correct for motion and magnetic susceptibility interactions. Temporal misalignment between different slices of the functional data (acquired in interleaved Siemens order) was corrected following SPM slice-timing correction (STC) procedure [83, 84], which uses ‘*sinc’* temporal interpolation to resample each slice BOLD timeseries to a common mid-acquisition time. Potential outlier scans were identified using ART [85] as acquisitions with framewise displacement above 0.9 mm or global BOLD signal changes above 5 standard deviations [86, 87], and a reference BOLD image was computed for each subject by averaging all scans excluding outliers. Functional and anatomical data were normalized into standard MNI space, segmented into grey matter, white matter, and cerebrospinal fluid tissue classes, and resampled to 1 mm isotropic voxels following a direct normalization procedure [87, 88] using SPM unified segmentation and normalization algorithm [89, 90] with the default IXI-549 tissue probability map template. Functional data were smoothed using spatial convolution with a Gaussian kernel of 5 mm full width half maximum (FWHM).

### Denoising

In addition, functional data were denoised using a standard denoising pipeline [91] including the regression of potential confounding effects characterized by white matter timeseries and their first order derivatives (20 CompCor noise components), CSF timeseries and their first order derivatives (20 CompCor noise components), motion linear and quadratic parameters and their first order derivatives (24 factors) [92], outlier scans (below 30 factors) [86], session and task effects and their first order derivatives (12 factors), and linear trends (2 factors) within each functional run, followed by high-pass frequency filtering of the BOLD timeseries [93] above 0.008 Hz. CompCor [94, 95]. Noise components within white matter and CSF were estimated by computing the average BOLD signal as well as the largest principal components orthogonal to the BOLD average, motion parameters, and outlier scans within each subject’s eroded segmentation masks. From the number of noise terms included in this denoising strategy, the effective degrees of freedom of the BOLD signal after denoising were estimated to range from 240.1 to 274.9 (average 271.2) across all subjects [87].

### Quality assessment (QA) of the denoised data

Quality control (QC) measures were performed for all 78 subjects during both fMRI sessions to control for noise confounds and ensure the validity and suitability of the resulting fMRI data following denoising steps for FC analyses. Data were examined for (1) QA denoising: distribution of quality control-functional connectivity (QC-FC) associations; (2) density distribution of FC values; and (3) subject level QC distribution levels for exclusion of outlier subjects. QC-FC%; a percent match in QC-FC correlations which is a measure property of the entire dataset for the distance between the observed distributions of correlations across participants for individual QC measures and FC strength. QC-FC% match levels above 95% indicate negligible modulations in the BOLD signal correlation associated with nuisance factors [96]. We calculated these measures for invalid scans, proportional valid scans (PVS) and mean motion. Whilst our QC-FC% was closer to 95%, each of the individual parameters had 95% overlap with the null hypothesis (NH). FC density distribution, which is the within-run FC strength distribution estimate (r coefficients) was calculated between all pairs of 1000 randomly selected voxels from functional runs of the entire data (*n = 78*). After denoising, all subject-level QC measures were estimated using preprocessed functional (Invalid scans, PVS, Mean Motion, Norm_func_) data, preprocessed anatomical - MNI-space template to anatomical overlap (Norm_anat_), and denoised functional (degrees of freedom [DOF], standard deviation of BOLD signal [BOLDstd], and GCOR [global correlation]) data. Extreme outlier identification was performed as values 3IQR (interquartile range) (below the 1^st^ quartile or above the 3^rd^ quartile – red dotted lines in Fig. 1B,C). Mild outliers were defined as values of 1.5 IQR i.e. below the 1^st^ quartile or above the 3^rd^ quartile (red-dashed lines), Fig. 1D. Out of 78 participants, 10 were marked as outliers and excluded from further analysis. Final statistical analysis was performed on the remaining 68 participants: 17 pwLC, 27 pwME/CFS and 24 HC.

### Statistical analysis

ROI-to-ROI connectivity matrices were estimated to characterise the patterns of FC with 67 ROIs. Connectivity strength was represented by Fisher-transformed bivariate correlation coefficients from a weighted general linear model (weighted-GLM) [97] which was defined separately for each pair of seed and target areas to model associations between their BOLD signal timeseries. Individual scans were weighted by a boxcar signal characterizing each task/experimental condition convolved with an SPM canonical haemodynamic response function and rectified.

Group differences were estimated for 68 subjects using a GLM [98]. For each individual voxel, a separate GLM was estimated, with first-level connectivity measures at this voxel as dependent variables (one independent sample per subject and one measurement per experimental condition, if applicable), and groups or other subject-level identifiers as independent variables. Voxel-level hypotheses were evaluated using multivariate parametric statistics with random-effects across subjects and sample covariance estimation across multiple measurements. Inferences were performed at the level of individual clusters i.e., groups of contiguous voxels. Cluster-level inferences were based on parametric statistics from Gaussian Random Field theory [97, 99]. Results for 68 subjects were thresholded using a combination of a cluster-forming *p < 0.001* voxel-level threshold, and a familywise corrected *pFDR < 0.05* cluster-size threshold [100].

## Results

### Within-group differences - healthy controls (post > pre)

**Fig. 2:**
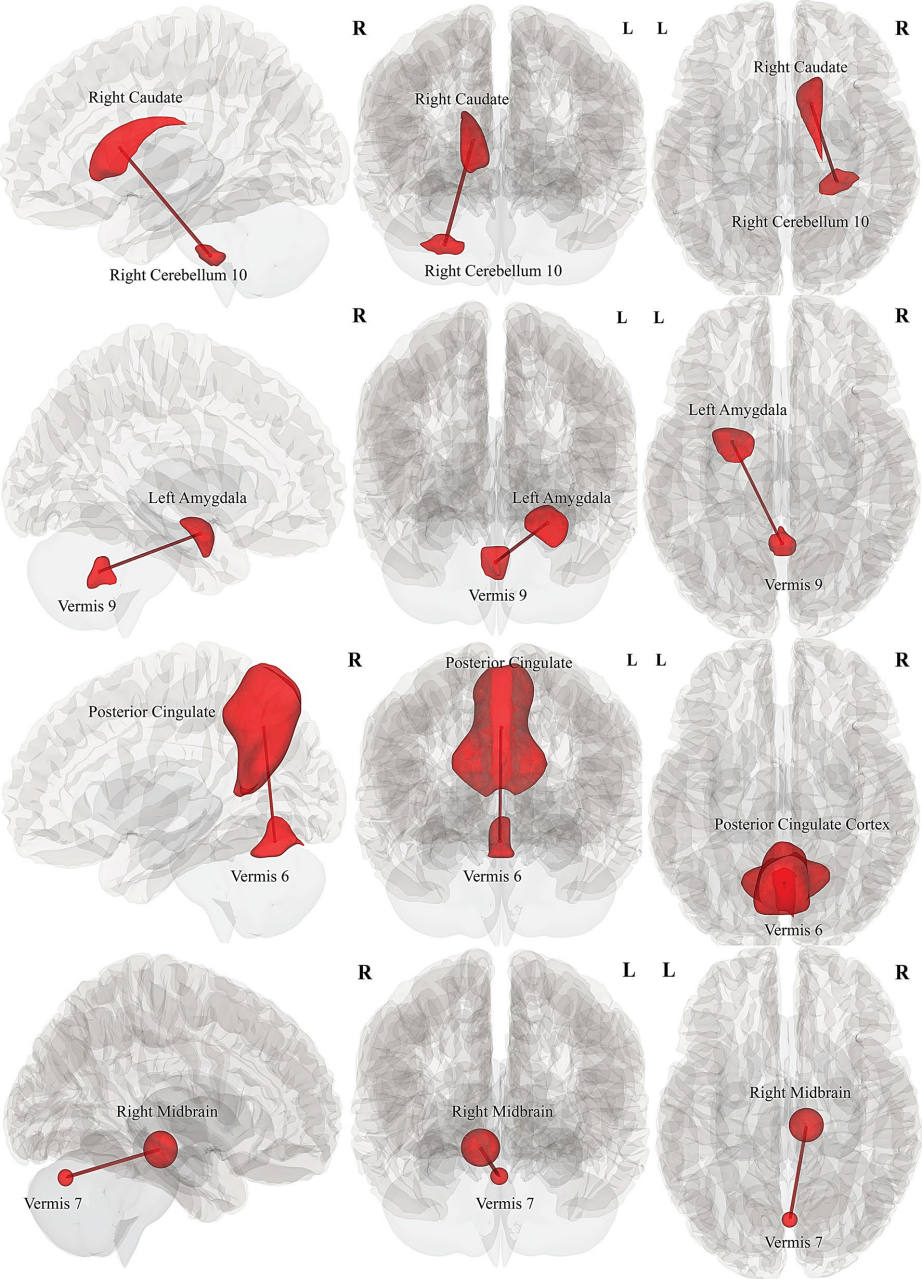
Illustration of FC differences within HC between *Post and Pre. *Rows from top to bottom: increased FC between right cerebellum 10 and right caudate nucleus seeds, increased FC between left amygdala and vermis 9; increased FC between posterior cingulate cortex and vermis 6; increased FC between right midbrain and vermis 7; *(left to right: sagittal, anterior and superior view)*

For differences between *Pre* and *Post* conditions, significantly increased FC or hyperconnectivity (in red) was observed in 24 healthy controls during the cognitively fatiguing *Post* condition. This increased FC was observed between the right nucleus caudate and right cerebellum 10 (*p = 0.04);* left amygdala and vermis 9 *(p = 0.005);* vermis 6 and posterior cingulate cortex *(p = 0.03);* vermis 7 and right midbrain *(p = 0.009)*, (see Table 2 and Fig. 2).

**Table 2:** Intra-group and inter-group FC differences during *Post and Pre *conditions for HC, pwLC, and pwME/CFS with source and target ROIs, beta, T and p-values with increased or reduced connectivity types provided. Multiple comparisons were corrected for p-FDR < 0.05 and controlled for nuisance factors (age and sex)

Condition	Seed Source	Target	Beta	t-value	p-FDR	FC
**Intra-group FC**						
**HC**						
Post > pre	Right nucleus caudate	Right cerebellum 10	0.40	4.03	0.040	Increased
	Left amygdala	Vermis 9	0.61	4.83	0.005	Increased
	Vermis 6	DMN.PCC	0.54	4.14	0.030	Increased
	Vermis 7	Right midbrain	0.60	4.63	0.009	Increased
**pwLC**						
Post > pre	Left nucleus accumbens	Vermis 3	0.39	4.62	0.026	Reduced
	DMNmnPFC	Left hippocampus	−0.41	−4.64	0.025	Increased
**pwME/CFS**						
Post > pre	Left nucleus cuneiform	Right medulla	−0.41	−3.97	0.037	Increased
**Inter-group FC**						
**pwLC > HC**						
Pre	Left amygdala	Vermis 7	−0.39	−3.80	0.036	Reduced
	Right cerebellum 9	Anterior cingulate cortex	−0.60	−4.12	0.014	Reduced
	Left medulla	Left hippocampus	−0.44	−3.79	0.037	Reduced
Post	Left nucleus accumbens	Vermis 3	−0.55	−4.82	0.001	Reduced
	Right cerebellum 7b	Vermis 6	0.45	3.93	0.025	Increased
**pwME/CFS > HC**						
Pre	Left LP	Right rPFC	−0.96	−3.57	0.042	Reduced
		Vermis 7	−0.55	−3.44	0.042	Increased
	Right rPFC	PCC	−0.92	−3.60	0.028	Increased
		Left LP	−0.96	−3.57	0.028	Reduced
Post	Right thalamus	Left midbrain	0.50	3.71	0.037	Increased
	Right cerebellum 7	Vermis 6	0.47	3.74	0.03	Increased
**pwLC > pwME/CFS**						
Pre	PCC	Vermis 9	−0.48	−3.87	0.027	Increased
Post	Right thalamus	Right putamen	0.45	3.70	0.04	Increased
	Left putamen	Left thalamus	0.49	3.37	0.04	Increased
		Left amygdala	0.40	3.32	0.04	Increased
		PCC	0.48	3.32	0.04	Reduced

*LP = left lateral parietal cortex; rPFC = right prefrontal cortex; PCC = posterior cingulate cortex; DMN.mPFC = medial prefrontal cortex of the DMN*

### Within-group differences - pwLC (post > pre)

**Fig. 3:**
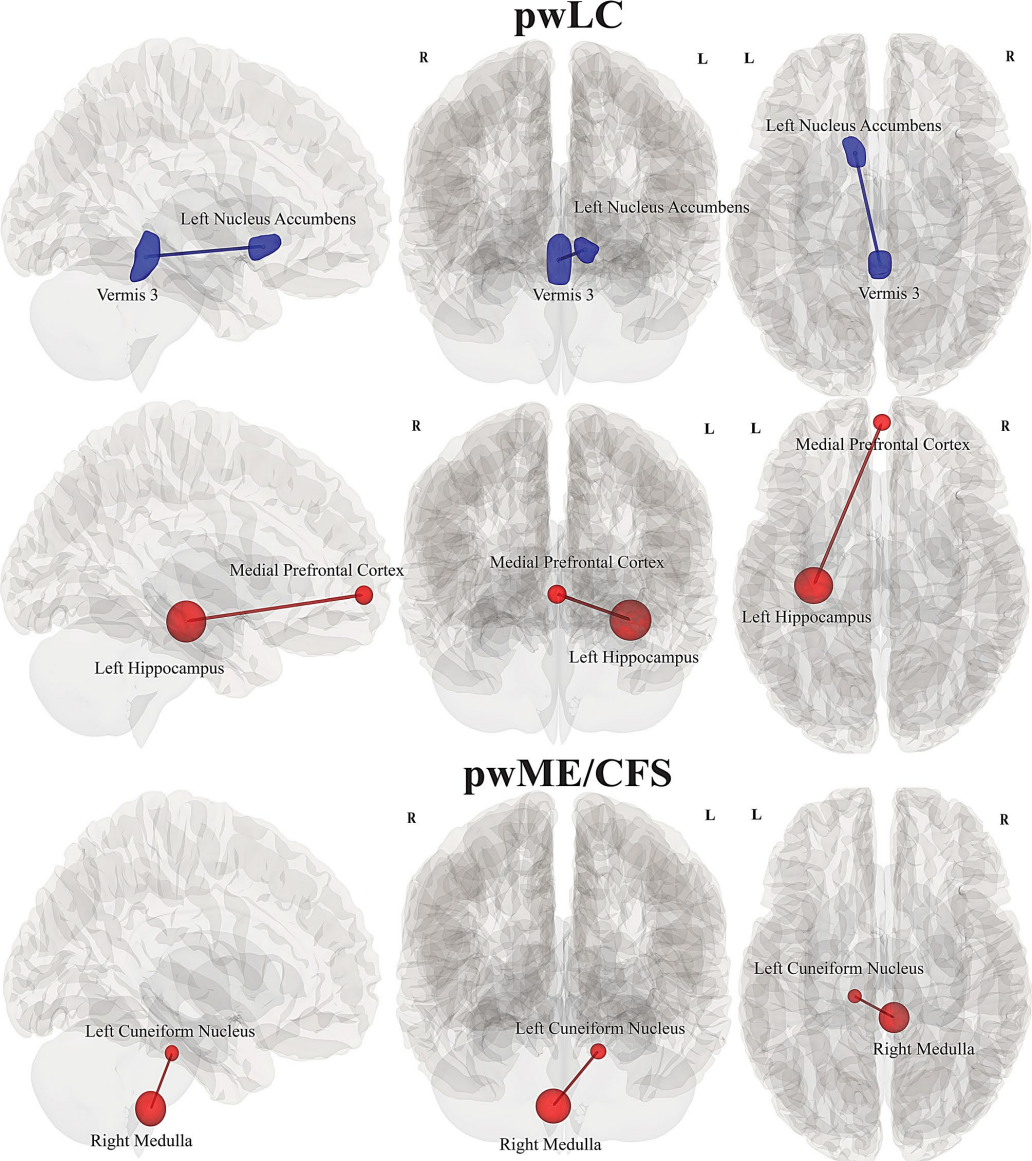
Illustration of FC differences within pwLC and pwME/CFS between *Post and Pre*. Rows from top to bottom: decreased FC between left nucleus accumbens and vermis 3; increased FC between medial prefrontal cortex and left hippocampus. For pwME/CFS, increased FC between brainstem left cuneiform nucleus and right medulla; *(left to right: sagittal, anterior and superior view)*

Reduced FC or hypoconnectivity (in blue) was significant for 17 pwLC during *Pre* between left nucleus accumbens and vermis 3 *(p = 0.02).* Increased FC (in red) was significant during *Post* between regions of medial prefrontal cortex (mPFC) and left hippocampus *(p = 0.02)*, (see Table 2, Fig. 3).

### Within-group differences - pwME/CFS (post > pre)

Increased FC was significant in 27 pwME/CFS between left cuneiform nucleus and right medulla *(p = 0.037)* during *Post* condition. while no significant connectivity was observed in *Pre*, (see Table 2, Fig. 3).

### Between-group FC differences for effects in *pre* and *post*

#### pwLC vs HC

**Fig. 4:**
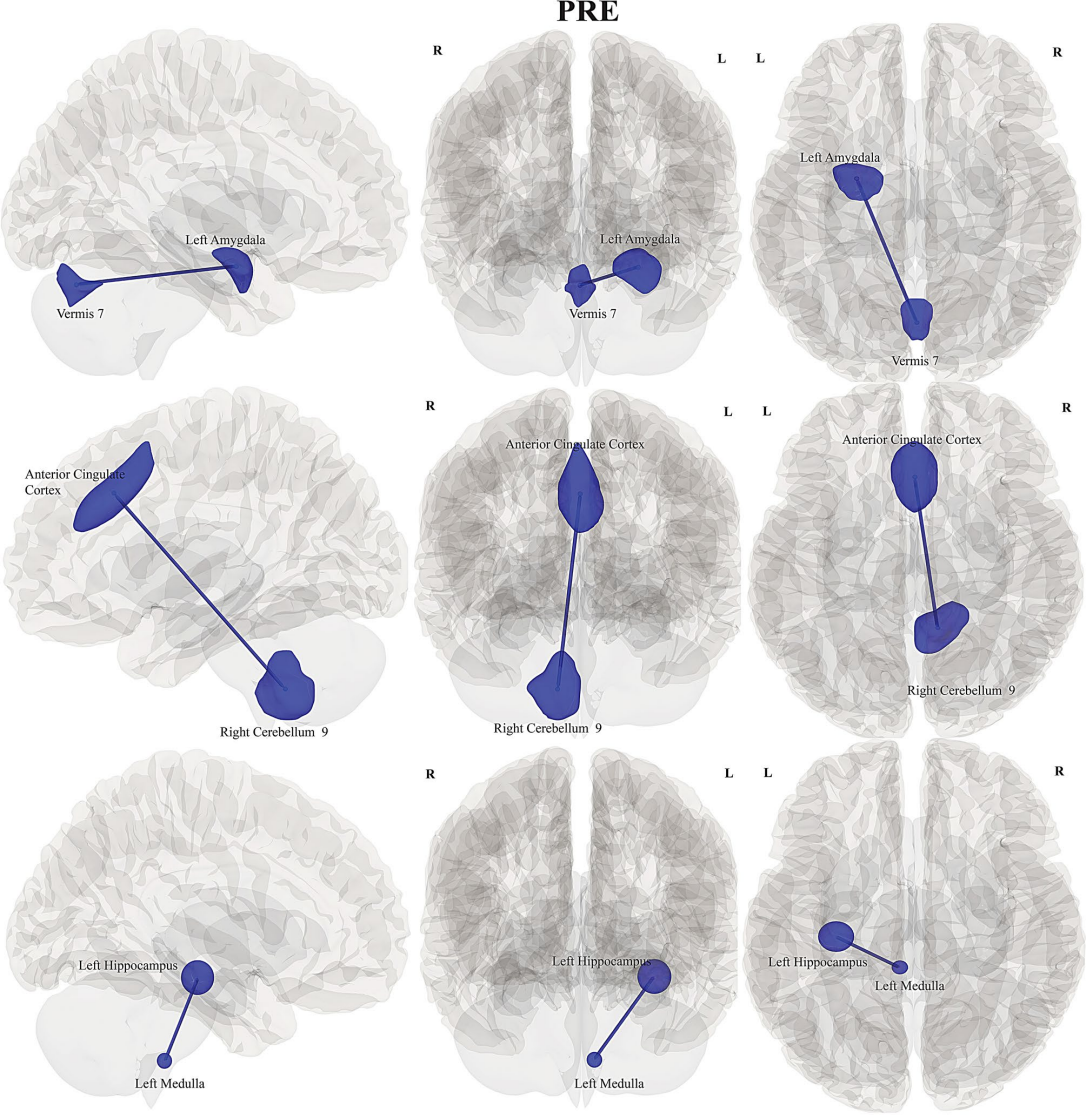

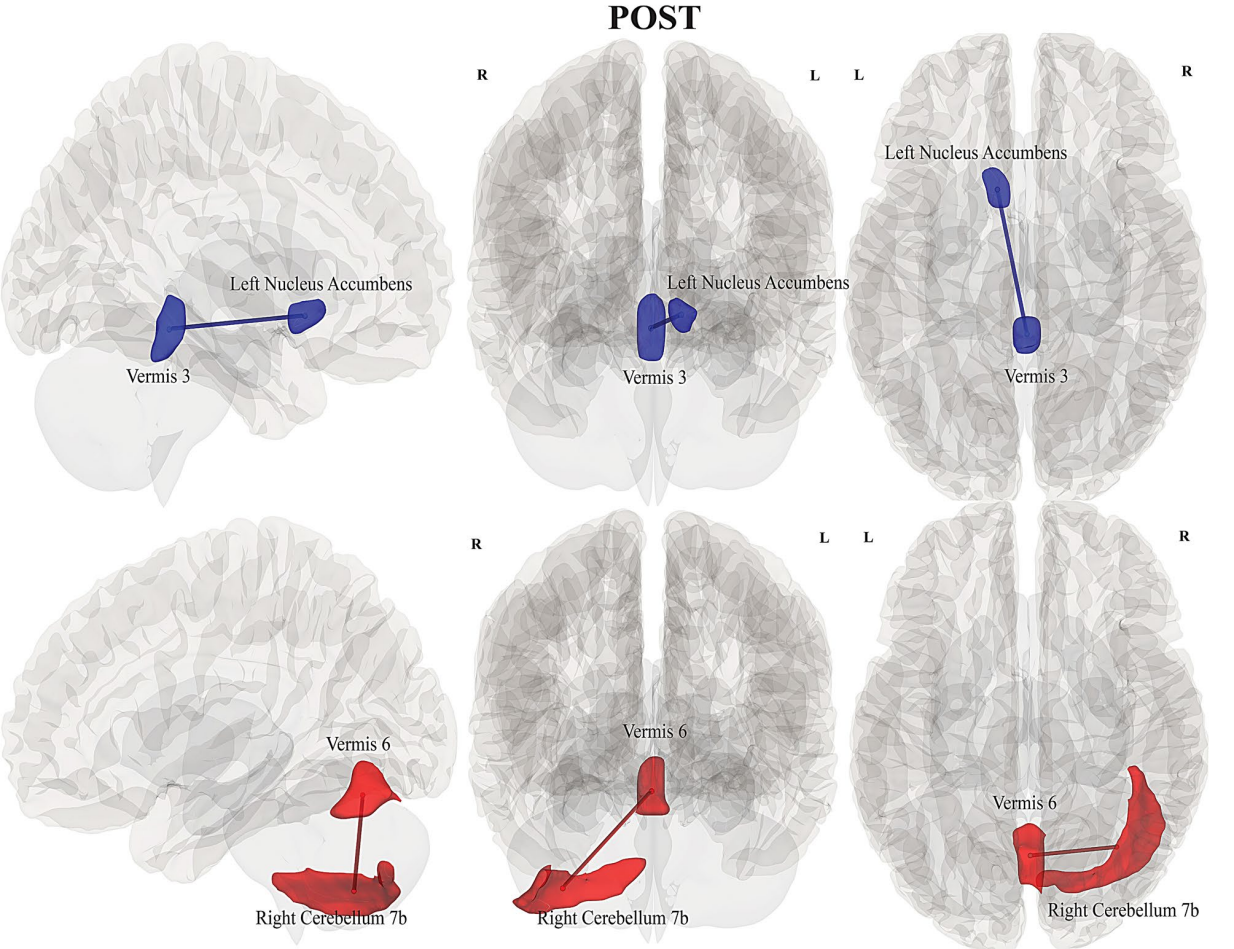
Illustration of between-group FC differences of pwLC and HC, for *Pre* and *Post* conditions. Rows from top to bottom: pwLC showed reduced FC between left amygdala and vermis 7, decreased FC between anterior cingulate cortex and right cerebellum 9; reduced FC between left hippocampus and left medulla. During *Post*, pwLC had reduced FC between left nucleus accumbens and vermis 3, while HC had increased FC between vermis 6 and right cerebellum 7b; *(left to right: sagittal, anterior and superior view)*

Significantly reduced FC in pwLC during *Pre* was observed between regions of left amygdala and vermis 7 (*p = 0.03)*; right cerebellum 9 and anterior cingulate cortex *(p = 0.01)*; left medulla and left hippocampus (*p = 0.04*), (see Table 2, Fig. 4). pwLC also showed reduced FC between left nucleus accumbens and vermis 3 *(p = 0.001)* during *Post*. In contrast, HC showed increased FC association between right cerebellum 7b and vermis 6 *(p = 0.02)*.

#### pwME/CFS vs HC

Complex FC connections were significant for pwME/CFS when compared with HC during *Pre.* Increased FC was significant between right rostro-prefrontal cortex (rPFC) hub of salience network and posterior cingulate cortex (PCC) (*p = 0.028*); left lateral parietal (LP) of the default mode network and vermis 7 (*p* = 0.04); whereas reduced FC was significant between rPFC and left LP, (*p* = 0.028 & *p* = 0.04).

**Fig. 5:**
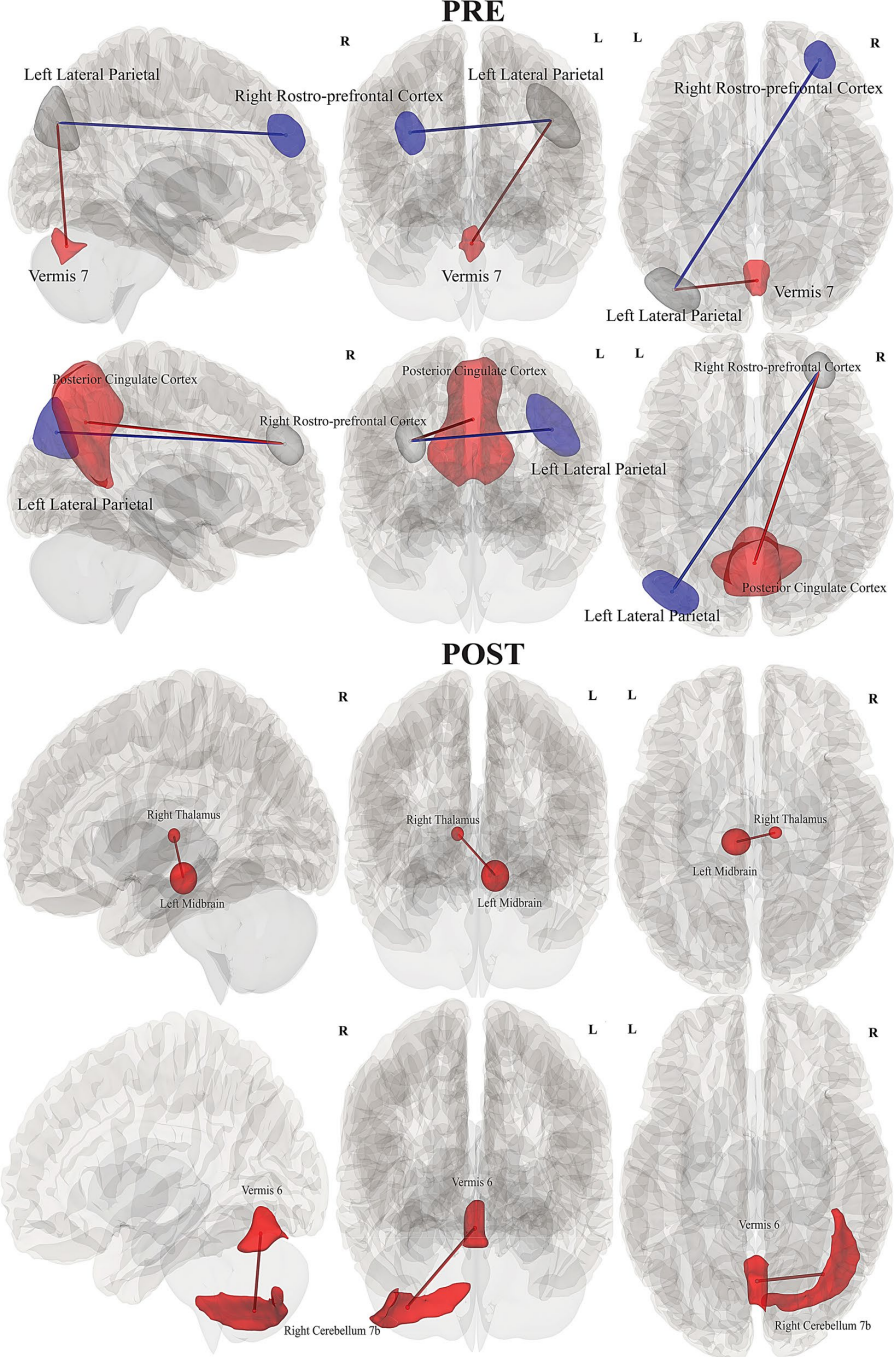
Illustration of between-group FC differences between pwME/CFS and HC, for *Pre* and *Post* conditions. Rows from top to bottom: reduced FC was significant in pwME/CFS, between right rostro-prefrontal cortex and left lateral parietal, and increased FC with posterior cingulate cortex; left LP also showed increased FC with vermis 7. Increased FC was significant in HC during *Post* for right thalamus with left midbrain; right cerebellum 7 and vermis 6 in HC. *(left to right: sagittal, anterior and superior view)*

During *Post* increased FC was significant in HC between right thalamus and left midbrain *(p = 0.037);* between right cerebellum 7 and Vermis 6 *(p = 0.03)*, (see Table 2, Fig. 5).

#### pwLC vs pwME/CFS

**Fig. 6:**
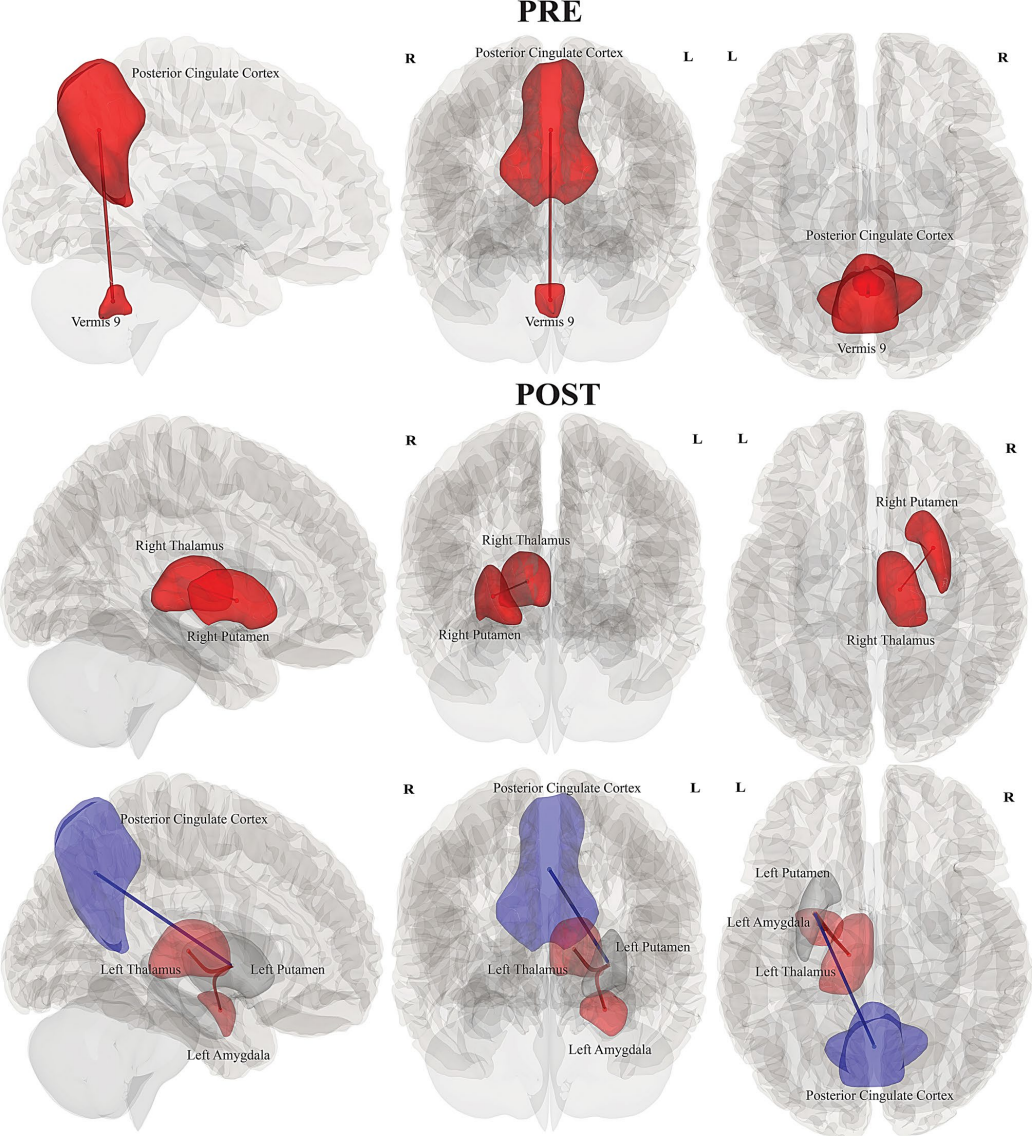
Illustration of between-group FC differences of pwME/CFS and pwLC for *Pre* and *Post* conditions. Rows from top to bottom: pwME/CFS showed increased FC during *Pre* for PCC and vermis 9. pwLC showed increased FC during *Post* in bilateral thalamus and putamen; left putamen showed increased FC with left amygdala and reduced FC with the PCC. *(left to right: sagittal, anterior and superior view)*

Increased connectivity was significant in pwME/CFS during *Pre* for FC differences between pwME/CFS and pwLC among regions of PCC and vermis 9 (*p = 0.02*). pwLC did not show any significant connectivity in *Pre*, however subcortical increased connections were significant during *Post* in pwLC, involving bilateral thalamus and putamen (*p = 0.04*); whereas left putamen showed hyperconnectivity with left amygdala and reduced connectivity with the PCC (*p = 0.04*), (see Table 2, Fig. 6).

### Correlation with clinical scores: duration of illness and cognitive scores

We tested for FC associations with ME/CFS and long COVID clinical measures for any effects during *Pre* and *Post* conditions. Significant connectivity values were extracted using CONN’s calculator tool and Pearson’s correlations were calculated in SPSS to estimate the strength of the correlations. The duration of illness for pwME/CFS positively correlated during *Pre* for right hippocampus and vermis 4 & 5 (*p = 0.003, r = 0.576*), and cerebellum 6 (*p= < 0.001, r = 0.700*). A significant negative correlation was detected between right hippocampus and vermis 4 & 5 during *Post* (*p = 0.004, r = −0.556*). However, no FC association was significant for duration of illness in pwLC. Furthermore, we tested FC correlations with cognitive deficit scores in pwME/CFS, where a negative correlation was significant for FC between cerebellum 6 and left caudate nucleus (*p= < 0.001, r = −0.802*) during *Post*; and between left SMG seed and amygdala (*p= < 0.001, r = −0.731*) during *Pre.* Likewise, pwLC also showed positive FC association between right SMG seed and left cerebellum 7 (*p = 0.001, r = 0.793*); and left red nucleus (*p = 0.001, r = 0.786*) during *Pre* condition for cognitive scores. *See* Supp. Figs 2–8*for FC correlations with the clinical scores*.

## Discussion

We report ROI-to-ROI functional connectivity differences between the cortical and subcortical brain regions in pwLC, pwME/CFS and HC using baseline and repeat 7T task fMRI. We found regional connectivity to be significantly disparate between patient and healthy cohorts secondary to cognitive fatigue induced by a mental exertion task. Significantly reduced FC was a prevalent feature in pwLC and pwME/CFS while HC showed characteristic increased connectivity among regions during repeat-task fMRI.

### Intra-group functional connectivity differences

Within-group FC analysis revealed HCs demonstrating an increased or positive connectivity between subcortical regions of the vermis, cerebellum, the midbrain, nucleus caudate of the basal ganglia and amygdala. Physiologically, these regions are highly interconnected with complex organisation forming centres for emotions, memory, attention, and are critical in sensory-motor pathways [101, 102]. This signifies the presence of a highly coordinated neural activity during task performance in healthy controls. These observations validate functional integration and network stability during processing of a cognitively demanding task in healthy participants. Contrarily, such hyperconnectivity was inconsistent in both ME/CFS and long COVID cohorts. Reduced or decreased connectivity between the nucleus accumbens and cerebellar vermis in pwLC was significant. These regions organise to form the cerebellum-amygdala-accumbens pathway, which is integral to reward-processing [103], driving the dopaminergic system in reward anticipation and motivation [104]. Negative association between these regions in our findings substantiate the presence of apathy in pwLC, i.e. a lack of interest and enthusiasm towards solving the energy-demanding cognitive exercise. Comparable observations were noted in Parkinson’s disease where reduced nucleus accumbens connectivity substantially correlated with reduced motivation [105]. The vermis is a region with diverse cognitive, motor and emotional processing roles [106, 107]. Reduced FC in this region, as observed in pwLC may possibly be due to increased apathy and reduced engagement during the Stroop task. Such outcomes have previously been documented with reduced FC between the nucleus accumbens, putamen, cerebellum, precuneus and insula, impacting decision making [104]. Increased FC between the hippocampus and mPFC is consistent with the neural activity between these two regions being frequently coordinated and synchronised in time [108]. The hippocampus serves to form and store memories as well as cognition while PFC is traditionally viewed as an executive hub for decision making, attention-set-shifting and working memory [109, 110]. Thus, increased connectivity correlations between the two regions in pwLC during the *Post*-interference condition may point towards an adaptive response to the complexity of the Stroop task. The act of recalling and imagining the future would also most likely result in increased communication between the two regions [111, 112]. These patterns were observed in a post-traumatic stress disorder (PTSD) study where patients with PTSD showed increased hippocampal-mPFC connectivity as an adaptive response to trauma [113]. Increased connectivity in pwME/CFS between cuneiform nucleus and medulla naturally imply high communication between neuronal projections running through the two regions which are critical for motor and autonomic functions via the medullary-reticulospinal tract. These results align with the observations reported by Barnden et. al., where rich hippocampal connections to both medulla and the midbrain cuneiform nucleus were reported in ME/CFS [58]. No sex-based FC differences were notable between the three cohorts. Nonetheless, further investigation is required to investigate subtle sex-based effects.

### Inter-group functional connectivity differences

When compared with HCs, pwLC displayed widespread reduction in FC across most subcortical regions, including amygdala, vermis-cerebellar network, medulla, hippocampus, nucleus accumbens, and anterior cingulate cortex (ACC) of the salience network. Intricate neural projections within these structures form highly organised pathways conjoining the ACC, and limbic regions of the amygdala, hypothalamus, thalamus, and brainstem which share distinct functionalities to network organisation [114]. Altogether, critical hubs for emotions, behaviour, memory and attention are established, enabling higher-order network topology [101]. The observed FC dynamics within these subcortical regions in pwLC explain the classic symptoms of emotional and cognitive deficits reported in long COVID. Current findings in concordance with observations reported by Baraniuk et al. [10] substantiate retroactive interference disruptions in pwLC [115].

A comparison of HCs with pwME/CFS revealed involvement of vermis, and nodes of the salience and the default mode networks in ME/CFS. The posterior cingulate cortex (PCC) is a DMN node and due to its heterogeneous nature, it is well known to be active when the brain is ‘freewheeling’ or at rest, during memory retrieval and regulating the focus of attention [116]. The rostro-prefrontal cortex (rPFC), also a DMN node, is noted to contribute towards mind wandering, spontaneous thoughts as alongside being involved in demanding tasks [117]. The lateral parietal cortex node of the DMN is also involved in cognitive functions, dedicated attention and working memory [118]. Synchronously, default mode network and deep cortical regions oversee cognitive information processing, learning and memory consolidation [73]. Compensatory activation in cerebellar vermis has been attributed to increased cognitive decline in Parkinson’s disease [119]. Hence, we may reason here that such interactions in ME/CFS reveal complex higher-level cognitive control that may be affected and/or influenced by the disease state, acute stress, or long-term compensatory mechanism [120, 121]. These regions also reportedly showed reduced glucose metabolism in positron emission tomography (PET) study in long COVID [122]. Viewed in aggregate, the evidence suggests that functional impairments and cognitive dysfunction may arise from reduced energy metabolism and present as executive and memory problems. Concurrently, the midbrain region that bridges the pons, thalamus and hypothalamus is vital in sensory and motor control pathways [102] and has been documented to play a role with the lateral parietal area in pwLC [34]. All in all, these intricate connections to paralimbic and limbic structures attest PCC and DMN as key players in information processing [123] that are compromised in ME/CFS.

These distinct FC observations can further be bolstered through multimodal imaging findings where reports of neurochemical imbalances in ME/CFS highlighted altered levels of choline, N-acetyl-aspartate (NAA), and lactate in right hippocampus [124], white matter [125], ACC [126, 127] and glutamate within the PCC in long COVID and ME/CFS [128]. Our ROI-to ROI functional connectivity results consolidate the involvement and alterations in these core regions which may be due to viral damage, low BOLD signals or perhaps a compensatory mechanism.

### FC correlations with illness duration and cognitive scores

Abnormal FC associations between cerebellum, vermis and hippocampus with the duration of illness in pwME/CFS suggest decreasing hippocampal-cerebellar communication over the disease course. FC values negatively correlating with an increase in the illness length suggests impairment with disease progression. More interestingly, this linear relationship was positive during *Pre* while a negative correlation was distinguishable during the fatiguing *Post* condition. This might possibly be due to reduced CBF to these regions expressed as reduced FC. Reduction in blood flow between these regions has also been linked with severe sleep and emotional disorders leading to depression [129]. In ME/CFS, this may as well be expressed by compensatory mechanisms as the disease progresses with age. Contrarily, no association was observed for illness duration and FC in long COVID patients which could be related to a much shorter disease course in pwLC (*mean = 0.8 yrs±0.7*) compared to ME/CFS patients (*mean = 12.4 yrs±11.2*).

In a comparable manner, complex FC correlations with WHODAS cognitive scores were significant for both pwLC and pwME/CFS, where associations were observed with the cerebellum, SMG, subcortical nucleus caudate and amygdala. In pwME/CFS, the involvement of the caudate, cerebellum and medullary reticular activating system or RAS in memory impairments is well-established [130]. Our results substantiate the findings that altered cerebellar-caudate FC correlates to impaired psychomotor vigilance, thereby implicating cerebellar involvement in fatigue-related cognitive processes [131]. Positive FC correlations in pwLC to the SMG, red nucleus, and cerebellum 7 are meaningful observations. The red nucleus is involved in movement control [132] for cognitive task engagement [133, 134] and thus, one can imply that increasing FC correlations between the red nucleus, cerebellum and salience network could be associated with and compensate for high level memory deficits in long COVID implicating red nucleus in cognitive dysfunction. However, further investigation is warranted.

In essence, peculiar connectivity patterns in ME/CFS show disparate functional connections which can be associated with the dysfunctional dynamic shifts during cognitively demanding tasks. Variability in connectivity patterns among brain regions between long COVID and ME/CFS cohorts may most likely reflect subtypes of the two conditions, with varying disease states, the course of illness, behavioural and biological abnormalities, resulting in a similar phenotypic presentation. While ME/CFS patients demonstrated complex recruitment of the DMN and SN nodes during cognitive fatigue, pwLC highly implicated subcortical deep grey matter regions during fatiguing task sessions. Furthermore, these essential brain networks failed dynamically to transition smoothly between exertional and functional states in ME/CFS and long COVID. Contrarily, healthy individuals consistently showed increased or hyperconnectivity between regions and networks in the repeat fMRI, thereby implying synchronous activity even in the presence of cognitive fatigue. Thus, we may conclude that FC driven by BOLD signals may be similar within the two patient groups, with changing metabolic and neural activity centred in DMN, cerebellum and limbic regions. FC stability and high resilience in HC provide a benchmark of cognitive efficiency in healthy populations indicating robust neural communication, while FC deficits in pwME/CFS and pwLC indicate disintegration and disorganisation between regions.

These FC abnormalities within and between cortical, subcortical and cerebellum regions can be associated with autonomic, attention, cognitive and fatigue symptomatology in long COVID and ME/CFS. A stark contrast between the two patient groups and healthy individuals is evident from this research, providing critical non-invasive network biomarkers. The dysfunctional networks in both syndromes pinpoint suitable targets for non-invasive modulation techniques. The use of ultra-high field 7T MRI, enhanced resolution, high signal-to-noise ratio and improved sensitivity permitted us to capture these subtle differences between long COVID and ME/CFS. fMRI certainly benefits from higher static magnetic field which leads to more reliable evaluation, and therefore clinical integration of mapping these disrupted network connectivity patterns can provide potential rationale for connectivity neuromodulation, by restoring network efficiency and symptom improvement in long COVID and ME/CFS.

## Limitations

This study lacks information regarding whether ME/CFS patients ever had COVID-19 infection, the number of times they were infected and whether ME/CFS patients received any formal diagnosis of long COVID comorbidity by a physician. Likewise, for long-COVID patients if they ever received an ME/CFS diagnosis. Present study also lacks categorising healthy controls following COVID-19 infection, i.e. COVID-recovered healthy controls, and those who have never contracted COVID infection. Clinical scores reported in this investigation were self-reported and some values were missing. However, we excluded the participants with missing values in our FC-correlation analysis. Group analyses for FC dynamics were not affected by the missing clinical scores. Nevertheless, despite small sample size, high sensitivity of 7T ensures robustness of these findings. Longitudinal studies are critical to fully comprehend the range and extent of symptomatology among the two patient cohorts.

## Conclusion

Characterisation of neural responses using functional connectivity analyses during a cognitively demanding exertion revealed differences within cortical and subcortical components highly implicated in long COVID and ME/CFS. Both long COVID and ME/CFS patients showed impaired functional connectivity among regions of the cerebellum, vermis, and deep grey matter during interference-induced cognitive fatigue which is indicative of cognitive exhaustion. This suggests reduced motivation and a decreased dopaminergic influence thereby inducing neural exhaustion. Furthermore, functional connections within the cerebellum and limbic regions correlated with the disease course in ME/CFS and cognitive scores in both patient groups. Nevertheless, patterns of aberrant functional connectivity in ME/CFS and long COVID patients may explain their shared symptomatology as a consequence of extensive pathological shifts across highly interconnected core networks regions.

## Electronic supplementary material

Below is the link to the electronic supplementary material.

Supplementary Material 1

## Data Availability

The raw data supporting the conclusions of this article will be made available by the authors upon reasonable request.
