# Supplementary material for: Distinct functional connectivity patterns in myalgic encephalomyelitis and long COVID patients during cognitive fatigue: a 7 Tesla task-fMRI study
Source: J Transl Med. 2026 Jan 20;24:236. doi: 10.1186/s12967-026-07708-y (PMC12903671; doi:10.1186/s12967-026-07708-y)
Supplement: Supplementary file 1 — Supplementary Material 1 [file 12967_2026_7708_MOESM1_ESM.docx]

**Supplementary Table 1.** *lists subjects with age, gender for health controls, ME/CFS, and long COVID patients included in the RRC analysis. Illness length (in years) is provided for patient cohorts with impaired WHODAS cognitive scores for all cohorts.* *NaN denotes not available.*

| **Subjects** | **Age** | **Sex** | **Duration of Illness** | **WHODAS Cognitive Score** |  |
| --- | --- | --- | --- | --- | --- |
| **HC (*n=24*)** | | | | | |
| 1 | 38.1 | 1 | 0 | 1 | |
| 2 | 33.2 | 2 | 0 | 1 | |
| 3 | 21.7 | 1 | 0 | 2 | |
| 4 | 36.5 | 2 | 0 | 1 | |
| 5 | 25.4 | 2 | 0 | 7 | |
| 6 | 43.3 | 1 | 0 | 10 | |
| 7 | 22.2 | 2 | 0 | NaN | |
| 8 | 50.1 | 1 | 0 | 8 | |
| 9 | 22.7 | 2 | 0 | 6 | |
| 10 | 47.1 | 1 | 0 | 0 | |
| 11 | 32.4 | 1 | 0 | 0 | |
| 12 | 33.5 | 1 | 0 | 5 | |
| 13 | 47.7 | 1 | 0 | 0 | |
| 14 | 43.4 | 2 | 0 | 4 | |
| 15 | 28.9 | 2 | 0 | 32 | |
| 16 | 40.7 | 1 | 0 | 0 | |
| 17 | 46.9 | 2 | 0 | 1 | |
| 18 | 35.2 | 2 | 0 | 4 | |
| 19 | 28.9 | 1 | 0 | 18 | |
| 20 | 25.9 | 1 | 0 | 2 | |
| 21 | 29.76 | 1 | 0 | 0 | |
| 22 | 28.1 | 1 | 0 | 2 | |
| 23 | 36.1 | 1 | 0 | 0 | |
| 24 | 36.3 | 1 | 0 | 2 | |
| **Long COVID (*n=17***) | | | | | |
| 25 | 49.1 | 1 | 0.75 | 35 | |
| 26 | 64.3 | 2 | 0.25 | 45 | |
| 27 | 45.5 | 1 | 0.17 | 20 | |
| 28 | 31.3 | 1 | 0.58 | 25 | |
| 29 | 40.8 | 2 | 0.16 | 50 | |
| 30 | 43.6 | 1 | 0.25 | 80 | |
| 31 | 62 | 2 | 0.33 | 50 | |
| 32 | 32 | 1 | 2 | 25 | |
| 33 | 19.6 | 1 | 0.5 | NaN | |
| 34 | 60.1 | 2 | 0.5 | 29.16 | |
| 35 | 52 | 1 | 1 | 12.5 | |
| 36 | 60 | 1 | 0.7 | 41.67 | |
| 37 | 60.6 | 1 | 0.5 | 25 | |
| 38 | 47.96 | 1 | 0.4 | 29.17 | |
| 39 | 38.38 | 1 | 1.7 | NaN | |
| 40 | 48.23 | 1 | 1.2 | 16.6 | |
| 41 | 30 | 2 | 2.7 | NaN | |
| **ME/CFS (*n=27***) | | | | | |
| 42 | 23.5 | 1 | 7 | 54.17 | |
| 43 | 25.6 | 1 | 2 | 50 | |
| 44 | 36.6 | 1 | 21 | 100 | |
| 45 | 45.3 | 1 | 10 | 25 | |
| 46 | 52.3 | 1 | 5 | 45.83 | |
| 47 | 38.5 | 1 | 12 | 45.83 | |
| 48 | 47.6 | 1 | 9 | 75 | |
| 49 | 30.3 | 2 | 2 | 58.3 | |
| 50 | 60.1 | 1 | 11 | 58.3 | |
| 51 | 39 | 1 | 25 | 41.67 | |
| 52 | 52.1 | 1 | 10 | 45.83 | |
| 53 | 44.1 | 1 | 8 | 54.17 | |
| 54 | 48.4 | 1 | 29 | 75 | |
| 55 | 45.6 | 1 | 24 | 50 | |
| 56 | 55.6 | 1 | 48 | 58.3 | |
| 57 | 59.8 | 1 | 12 | 54.17 | |
| 58 | 32 | 2 | 2 | 41.67 | |
| 59 | 46.5 | 2 | 8 | 45.83 | |
| 60 | 49 | 1 | 0.5 | 37.5 | |
| 61 | 30 | 1 | 1.9 | 50 | |
| 62 | 51.6 | 2 | 13 | NaN | |
| 63 | 43.6 | 2 | 24 | NaN | |
| 64 | 46.9 | 2 | 31 | 58.3 | |
| 65 | 32.3 | 1 | 8 | 50 | |
| 66 | 27.22 | 2 | 1.4 | NaN | |
| 67 | 36.64 | 1 | 8 | NaN | |
| 68 | 60.33 | 2 | 11.3 | NaN | |

**Supplementary Figure 1.** *Illustrates connectome of 67 regions of interest used in the RRC analysis for long COVID, ME/CFS and healthy controls.*


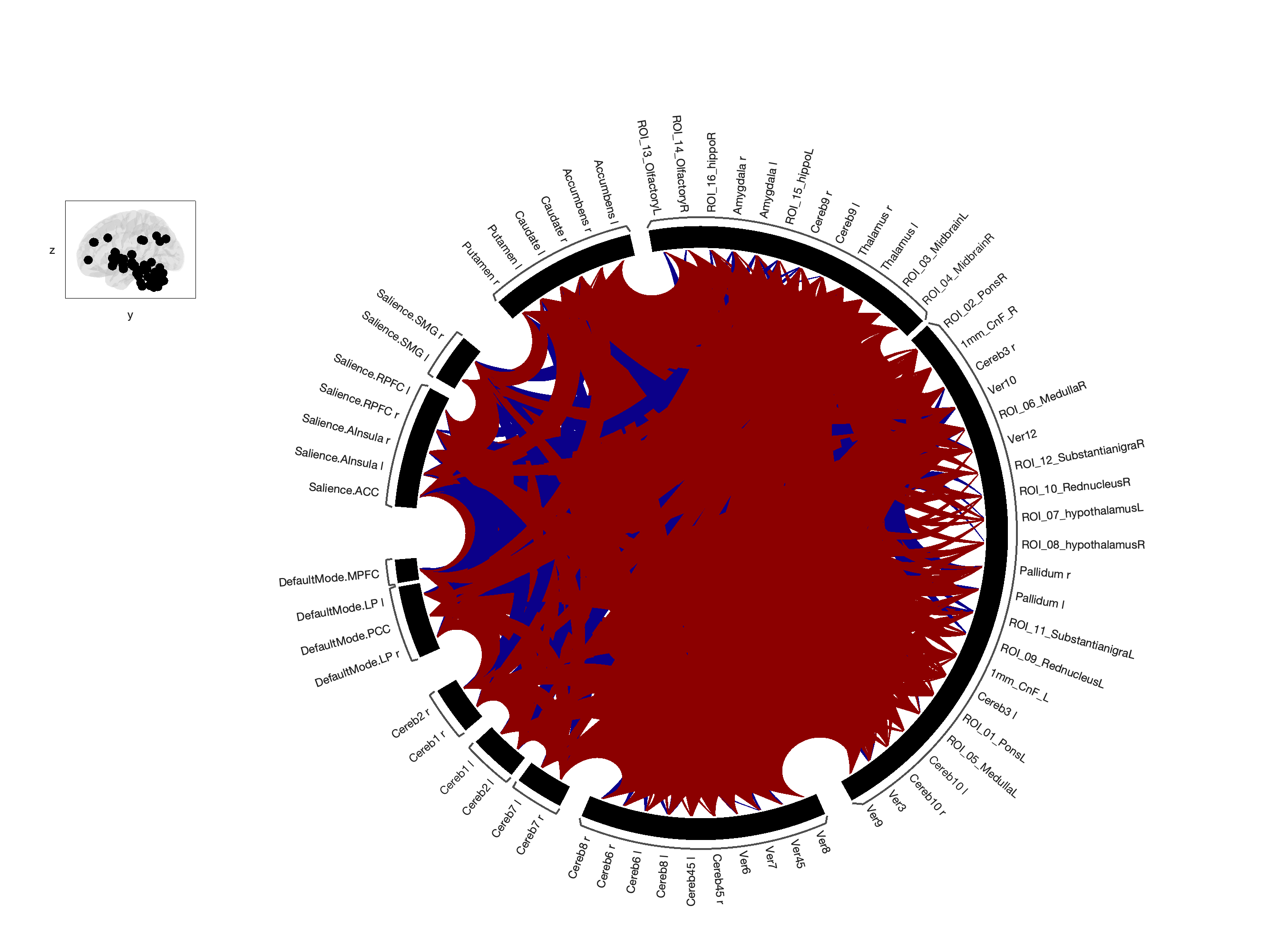


**Supplementary Table 2*.*** *List of ROIs used to test functional connectivity for patients with long COVID, ME/CFS and healthy controls.*

| **Regions of Interest** | **Abbreviations in Connectome** | |
| --- | --- | --- |
| **Default Mode Network** | | |
| Medial Prefrontal Cortex  Lateral Parietal  Posterior Cingulate Cortex | DefaultMode.MPFC  DefaultMode.LP (l/r)  DefaultMode.PCC | |
| **Salience network** | | |
| Rostro-PreFrontal Cortex  Supramarginal Gyrus  Anterior Cingulate Cortex  Anterior Insula | Salience.RPFC (l/r)  Salience.SMG (l/r)  Salience.ACC  Salience.AInsula (l/r) | |
| **Subcortical Regions** | | |
| Cerebellum Crus 1  Cerebellum Crus 2  Cerebellum 3  Cerebellum 4 & 5  Cerebellum 6  Cerebellum 7b  Cerebellum 8  Cerebellum 9  Cerebellum10  Vermis 1 & 2  Vermis 3  Vermis 4 & 5  Vermis 6  Vermis 7  Vermis 8  Vermis 9  Vermis 10  Thalamus  Nucleus Caudate  Nucleus Putamen  Pallidum  Nucleus Accumbens  Amygdala  Cuneiform Nucleus  Pons  Midbrain  Medulla  Hypothalamus  Red Nucleus  Substantia Nigra  Olfactory  Hippocampus | | Cereb1 (l/r)  Cereb2 (l/r)  Cereb3 (l/r)  Cereb4 5 (l/r)  Cereb6 (l/r)  Cereb7 (l/r)  Cereb8 (l/r)  Cereb9 (l/r)  Cereb10 (l/r)  Verm1 2  Ver3  Ver4 5  Ver6  Ver7  Ver8  Ver9  Ver10  Thalamus (l/r)  Caudate (l/r)  Putamen (l/r)  Pallidum (l/r)  Accumbens (l/r)  Amygdala (l/r)  1mm_CnF_L  1mm_CnF_R  ROI_01_PonsL  ROI_02_PonsR  ROI_03_MidbrainL  ROI_04_MidbrainR  ROI_05_MedullaL  ROI_06_MedullaR  ROI_07_hypothalamusL  ROI_08_hypothalamusR  ROI_09_RedNucleusL  ROI_10_RedNucleusR  ROI_11_SubstantiaNigraL  ROI_12_SubstantiaNigraR  ROI_13_OlfactoryL  ROI_14_OlfactoryR  ROI_15_hippoL  ROI_16_hippoR |

*L/R or l/r = left/right*

**Supplementary Figure 2.** Correlation is significant between FC values and duration of illness in pwME/CFS for vermis 4&5 and right hippocampus (*p=0.003*) during *Pre. x-axis shows distribution of duration of illness in years for pwME/CFS; y-axis shows FC values between seeds during Pre*

*
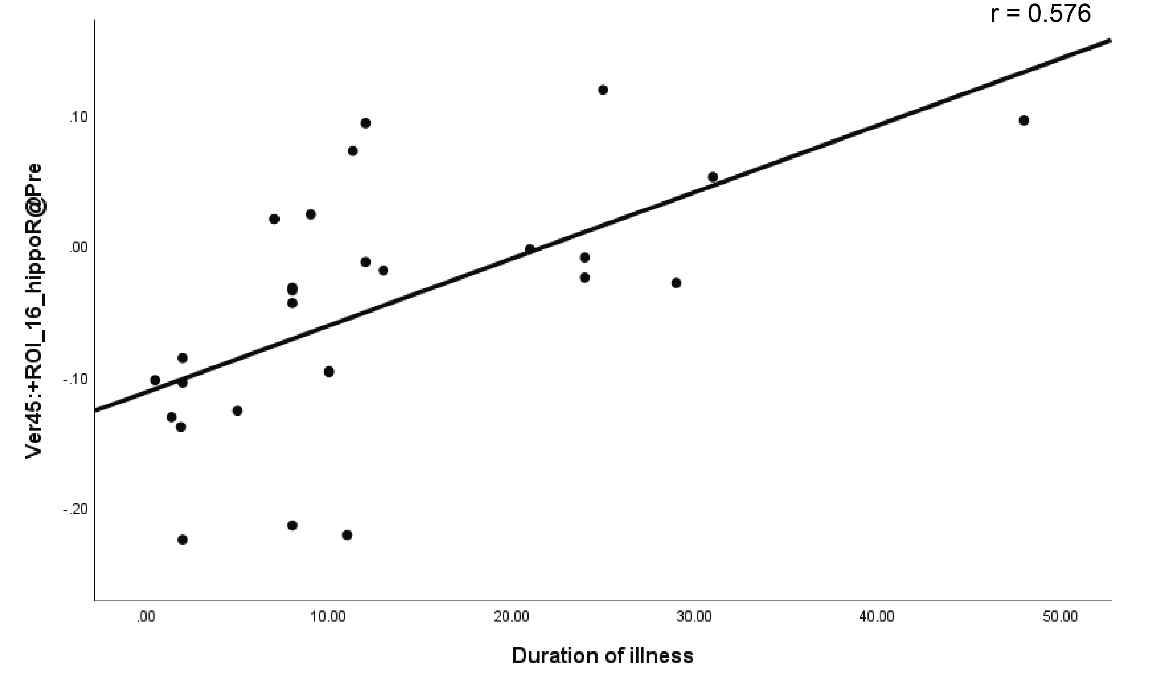
*

**Supplementary Figure 3.** Correlation is significant between FC values and duration of illness in pwME/CFS for right cerebellum 6 and right hippocampus (*p=<0.001*) during *Pre. x-axis shows distribution of duration of illness in years for pwME/CFS; y-axis shows FC values between seeds during Pre*


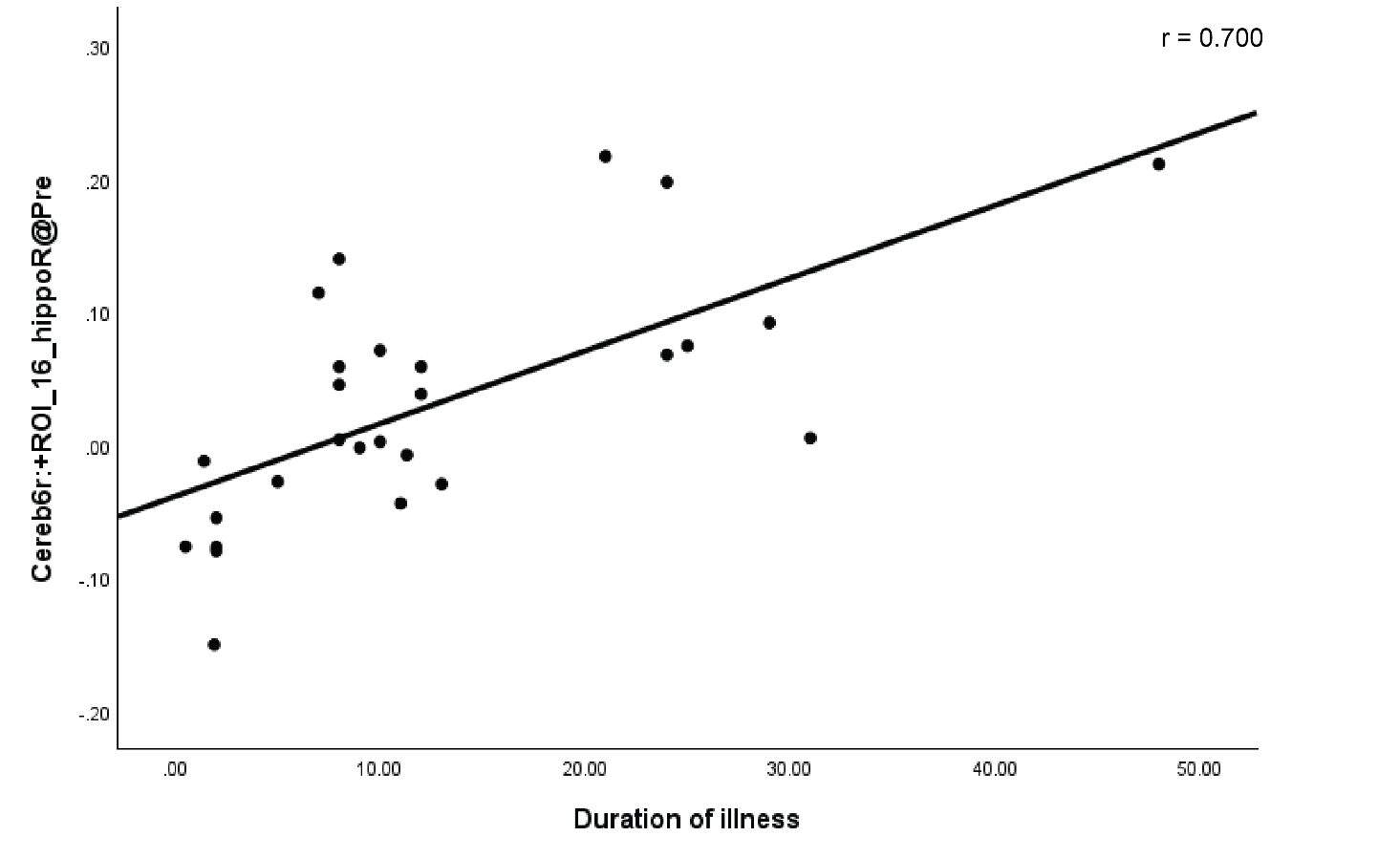


**Supplementary Figure 4.** Correlation is significant between FC values and duration of illness in pwME/CFS for vermis 4&5 and right hippocampus (*p=0.004*) during *Post. x-axis shows distribution of duration of illness in years for pwME/CFS; y-axis shows FC values between seeds during Post*


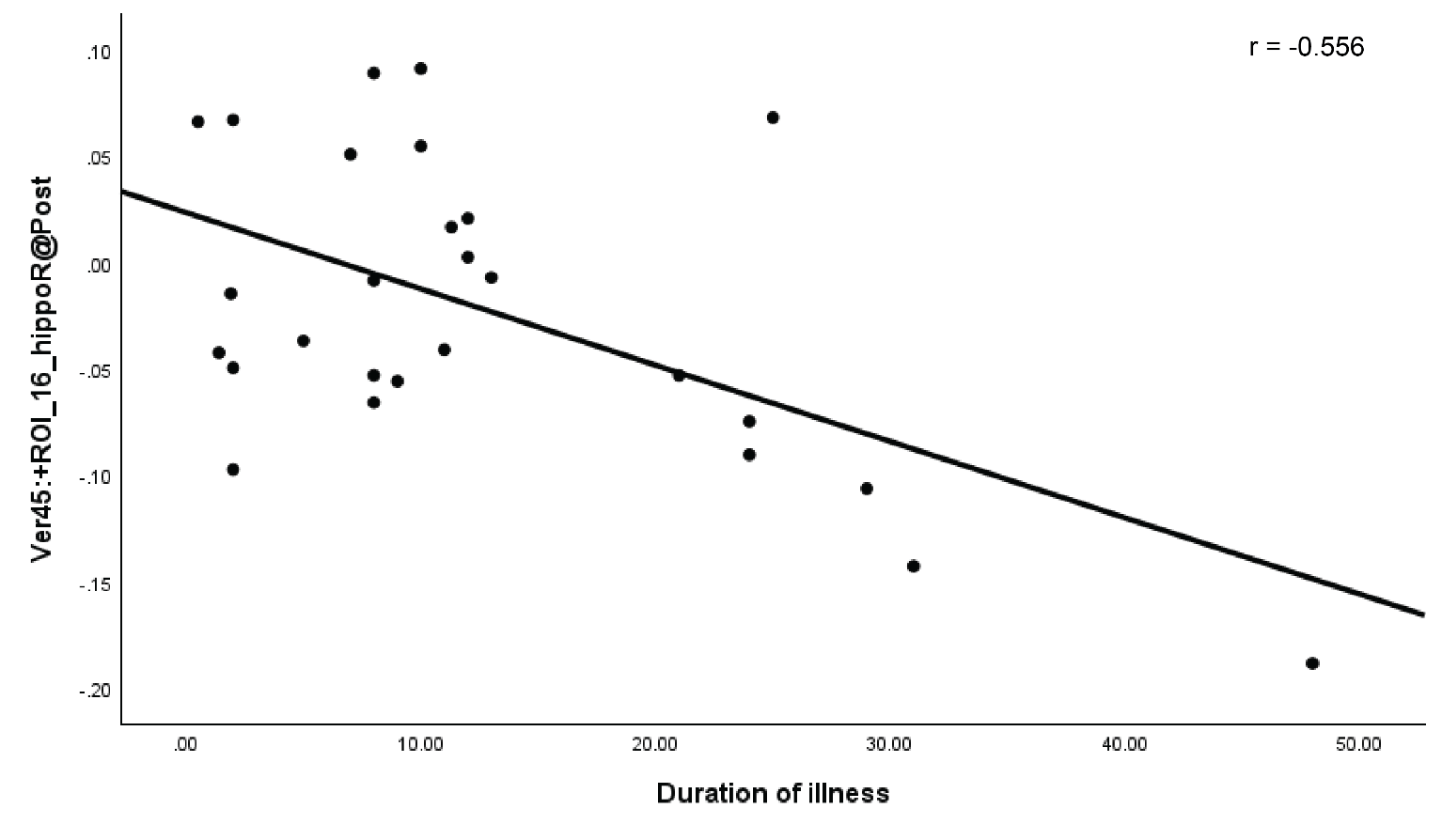


**Supplementary Figure 5.** Correlation is significant between FC values and cognitive scores in pwME/CFS for left caudate and cerebellum 6 right (*p=<0.001*) during *Post. x-axis shows distribution of WHODAS cognitive scores for pwME/CFS; y-axis shows FC values between seeds during Post*

*
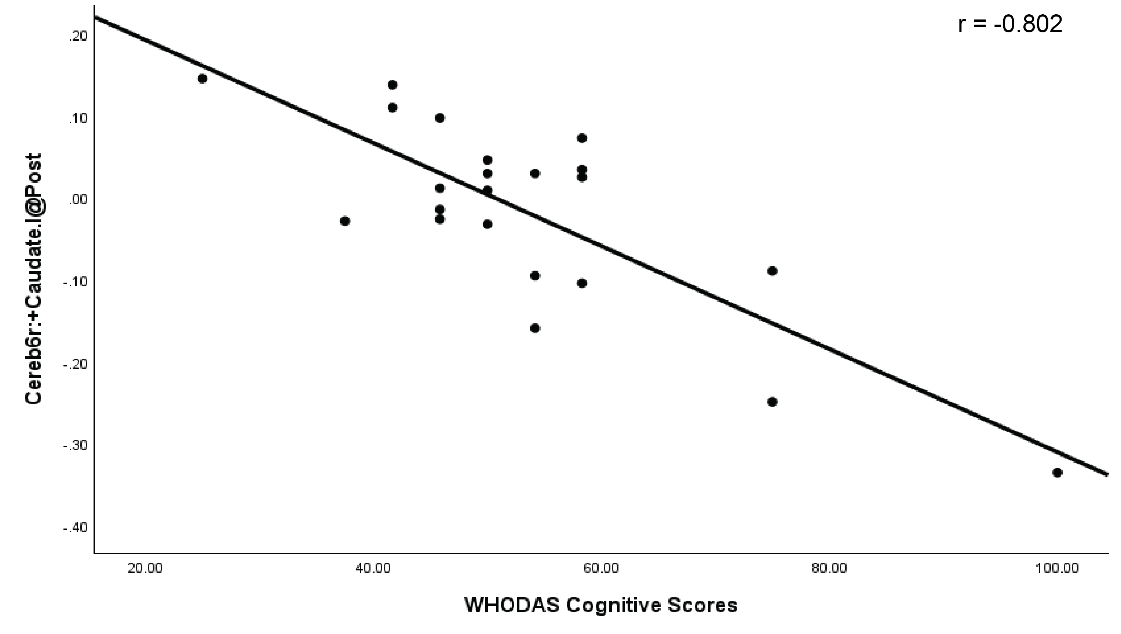
*

**Supplementary Figure 6.** Correlation is significant between FC values and cognitive scores in pwME/CFS for left SMG and right amygdala (*p=<0.001*) during *Pre. x-axis shows distribution of WHODAS cognitive scores for pwME/CFS; y-axis shows FC values between seeds during Pre*


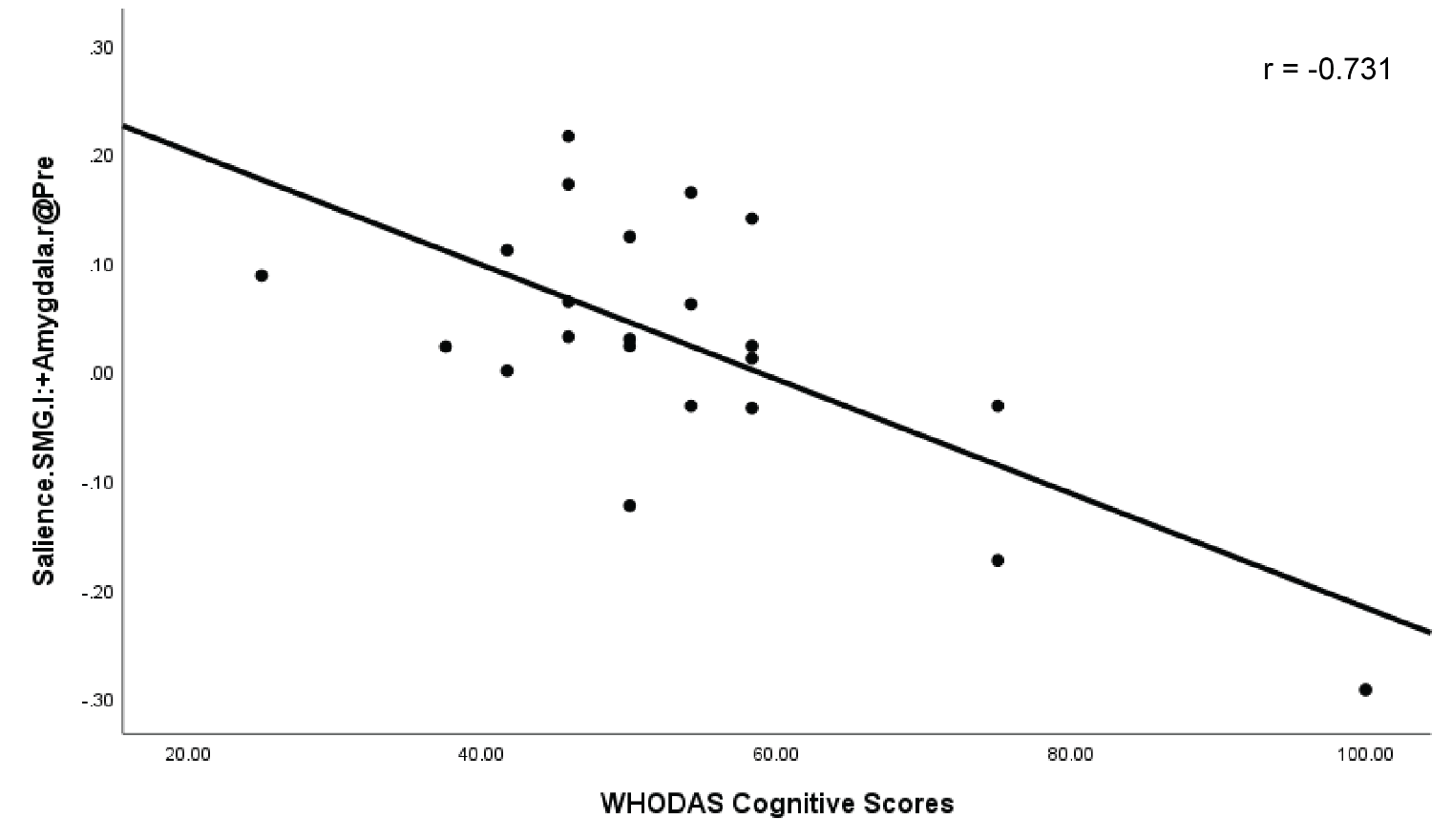


**Supplementary Figure 7.** Correlation is significant between FC values and cognitive scores in pwLC for left cerebellum 7 and right supramarginal gyrus (*p=0.001*) during *Pre. x-axis shows distribution of WHODAS cognitive scores for pwLC; y-axis shows FC values between seeds during Pre*


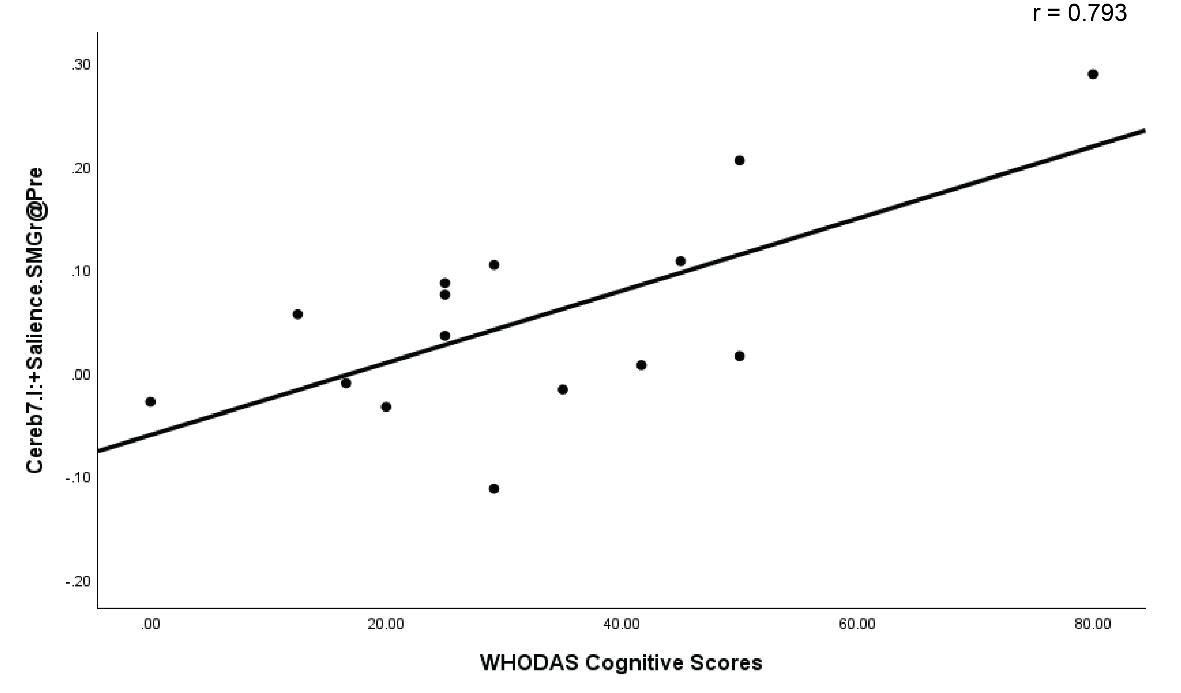


**Supplementary Figure 8.** Correlation is significant between FC values and cognitive scores in pwLC for left red nucleus and right supramarginal gyrus (*p=0.001*) during *Pre. x-axis shows distribution of WHODAS cognitive scores for pwLC; y-axis shows FC values between seeds during Pre.*

*
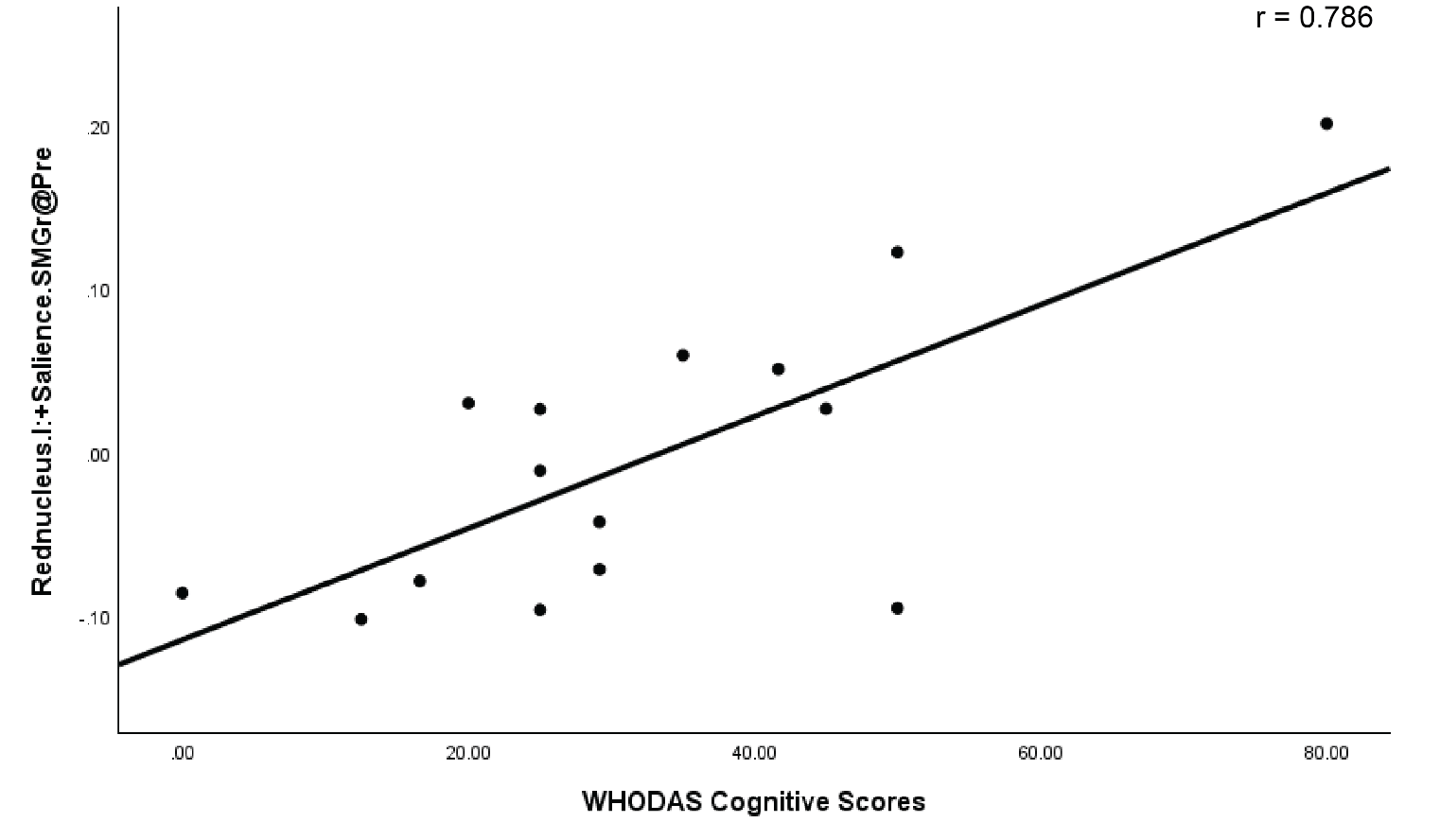
*
